# Siderophores and competition for iron govern myxobacterial predation dynamics

**DOI:** 10.1093/ismejo/wrae077

**Published:** 2024-05-02

**Authors:** Francisco Javier Contreras-Moreno, Aurelio Moraleda-Muñoz, Francisco Javier Marcos-Torres, Virginia Cuéllar, María José Soto, Juana Pérez, José Muñoz-Dorado

**Affiliations:** Departamento de Microbiología, Facultad de Ciencias, Universidad de Granada, E-18071 Granada, Spain; Departamento de Microbiología, Facultad de Ciencias, Universidad de Granada, E-18071 Granada, Spain; Departamento de Microbiología, Facultad de Ciencias, Universidad de Granada, E-18071 Granada, Spain; Departamento de Biotecnología y Protección Ambiental, Estación Experimental del Zaidín, CSIC, E-18008 Granada, Spain; Departamento de Biotecnología y Protección Ambiental, Estación Experimental del Zaidín, CSIC, E-18008 Granada, Spain; Departamento de Microbiología, Facultad de Ciencias, Universidad de Granada, E-18071 Granada, Spain; Departamento de Microbiología, Facultad de Ciencias, Universidad de Granada, E-18071 Granada, Spain

**Keywords:** Myxococcus xanthus, Sinorhizobium meliloti, iron competition, myxochelins, rhizobactin 1021

## Abstract

Bacterial predators are decisive organisms that shape microbial ecosystems. In this study, we investigated the role of iron and siderophores during the predatory interaction between two rhizosphere bacteria: *Myxococcus xanthus*, an epibiotic predator, and *Sinorhizobium meliloti*, a bacterium that establishes nitrogen-fixing symbiosis with legumes*.* The results show that iron enhances the motility of the predator and facilitates its predatory capability, and that intoxication by iron is not used by the predator to prey, although oxidative stress increases in both bacteria during predation. However, competition for iron plays an important role in the outcome of predatory interactions. Using combinations of predator and prey mutants (nonproducers and overproducers of siderophores), we have investigated the importance of competition for iron in predation. The results demonstrate that the competitor that, via the production of siderophores, obtains sufficient iron for growth and depletes metal availability for the opponent will prevail in the interaction. Consequently, iron fluctuations in soils may modify the composition of microbial communities by altering the activity of myxobacterial predators. In addition, siderophore overproduction during predation can alter soil properties, affecting the productivity and sustainability of agricultural operations.

## Graphical Abstract

**Graphical Abstract:**
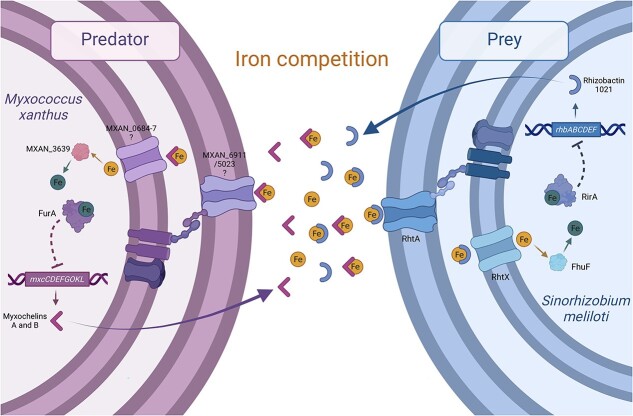

## Introduction

Micropredators shape and maintain the diversity of microbial communities, enhance plant health and yield, and influence food webs [1–9]. In aquatic and soil ecosystems, myxobacteria are major micropredators that play important ecological roles because they form a nexus between the interactions of all microorganisms in these habitats [10–12]. To kill and lyse other microorganisms, myxobacteria use an epibiotic group attack strategy [13]. In this study, we used the model myxobacterium *Myxococcus xanthus* as a predator, which employs a combination of weapons to prey, with some functioning at a distance and others requiring close contact; consequently, motility is also important during predation [14–22]. As prey, we used *Sinorhizobium meliloti*, a nitrogen-fixing bacterium that establishes symbiotic relationships with legumes and plays an important role in agricultural systems by enhancing soil fertility [23].

Most organisms need to maintain homeostasis for several metal ions because they are required for vital cellular functions but are toxic at high levels [24–26]. Therefore, predators might exploit this dual role to take advantage of the interaction, either by depleting essential metals for the prey or by intoxicating them with an excess. In protozoan predation, metals have been implicated in killing prey [27]. For instance, *Dictyostelium discoideum* uses Cu(I) to increase the amount of reactive oxygen species (ROS) inside the phagosome to kill bacteria [28]. However, studies on the implications of essential metals in bacterial predation are scarce. Copper has been studied in the predators *Cupriavidus necator* and *M. xanthus* [28, 29], and it could be hypothesized that predatory bacteria also use iron to prey.

The main mechanism used by bacteria for iron uptake is the production of siderophores, which are small and diverse molecules with a high affinity for ferric ions [31]. In Gram-negative bacteria, the uptake of iron–siderophore complexes (ferrisiderophores) is carried out by a transporter protein located in the outer membrane, termed TonB-dependent transporter (TBDT), because it is energized by the TonB system (Fig. S1) [31, 32]. Generally, the expression of genes involved in siderophore biosynthesis and ferrisiderophore transport is regulated by a repressor of the Fur (Ferric Uptake Regulator) family, although in some bacteria, such as rhizobia, the iron-responsive regulators are Irr and RirA [32]. To bind to the operator, Fur and RirA repressors require Fe(II), so that, in the absence of this metal, they are inactivated and genes under their control are expressed [31–35].

Although there is much information about siderophore production and uptake in several bacteria, the ecological role of these secondary metabolites remains unexplored. To date, studies on iron and predation have revealed that *M. xanthus* upregulated the expression of siderophores and depletes the iron supply to *Streptomyces coelicolor* [36]. These iron-restricted conditions are responsible for the overproduction of the antibiotic actinorhodin in prey [36, 37]. Other analyses have revealed that several myxobacterial predators (*M. xanthus, Cystobacter ferrugineus*) and prey (*S. meliloti*, *Micrococcus luteus*, *Escherichia coli, Pseudomonas putida*, and *Pseudomonas aeruginosa*) also upregulate the expression of genes involved in siderophore biosynthesis when they interact [38–42]. However, the role of iron and siderophores in the dynamics of bacterial predation has not been thoroughly addressed.

To investigate the impact of iron and siderophores on the predation of *M. xanthus* on *S. meliloti*, combinations of predator and prey mutants with altered siderophore production (either nonproducers or overproducers) and uptake were assayed. The results have revealed that the strain with the ability to get enough supply of iron and deplete the metal for the competitor will predominate. Therefore, the availability of iron will be decisive in the outcome of the interaction. As a result, siderophore overproduction during predatory interactions will have an impact on the soil microbiome and its properties.

## Materials and methods

### Bacterial strains, plasmids, and growth conditions

Bacterial strains and plasmids used in this study are listed in Table S1. *M. xanthus* strains were grown in CTT medium [43] at 30°C. When needed, 100 μg/ml X-gal (5-bromo-4-chloro-3-indolyl-β-D-galactopyranoside) and/or 220 μM FeCl_3_, to prevent the production of rhizobactin 1021 (Rz1021) [44], were added. *S. meliloti* strains were grown at 30°C in CTT, TY [45], or MM media [46]. Low-iron MM contained 2.2 μM FeCl_3_, and it was used for the selection of the *rirA* mutant. *E. coli* strains were grown in LB medium [47] at 37°C. Antibiotics were added, as appropriate, at the following final concentrations: for *M. xanthus*, kanamycin 80 μg/ml; for *S. meliloti*, streptomycin 200 μg/ml, kanamycin 200 μg/ml, neomycin 100 μg/ml, and tetracycline 10 μg/ml; and for *E. coli*, streptomycin 50 μg/ml, kanamycin 50 μg/ml, and tetracycline 10 μg/ml.

### Construction of the *M. xanthus* and *S. meliloti* mutants

Details of the strains used in this study are shown in Table S1. *M. xanthus* in-frame deletion mutants were generated using the pBJ113 vector [48] and the primers listed in Table S2. Plasmids (Table S1) were introduced into the *M. xanthus* wild type (Mx_WT) by electroporation to generate single mutants. To obtain the double mutant *mxcG_furA*, plasmid pFJCMΔmxcG (Table S1) was electroporated into the *furA* mutant. Strains were selected as previously described [49].

The in-frame deletion of the *S. meliloti rirA* gene was generated by overlap extension polymerase chain reaction (PCR) [50] using primers SmRirA1 to SmRirA4 (Table S2) and the suicide plasmid pK18 mobsacB, yielding plasmid pK18-ΔrirA (Table S1). This construction was introduced into the wild-type *S. meliloti* Rm1021 (Sm_WT) strain via conjugation by biparental mating using the *E. coli* mobilizing strain S17-1, and allele replacement events were selected as described previously [51]. To avoid the toxic effects caused by iron in putative *rirA* mutants, the last crossover event was selected in low-iron MM. The *rhtA* mutant was obtained by transferring the *rhtA::Tn5* mutation from strain 2011rhtA1 to Sm_WT by phage ΦM12 transduction [52]. Similarly, the *rhbA_rirA* mutant was obtained by transferring the *rhbA*::Tn*5lac* mutation from the *rhbA* mutant to the *rirA* mutant.

### Construction of *M. xanthus* and *S. meliloti* strains harboring *lacZ* fusions, and β-galactosidase assays

A plasmid harboring a fusion between the *M. xanthus mxcG* gene and *lacZ* was constructed using vector pKY481 [53] and the oligonucleotides listed in Table S2 as primers. The *Bam*HI site in the primer was introduced at the start codon of the *M. xanthus* gene and in frame with the *Bam*HI site existing in the *lacZ* gene of plasmid pKY481. This plasmid was introduced into *M. xanthus* by electroporation, and the strains were selected as previously described [49].

To obtain a transcriptional fusion of the *S. meliloti* promoter for the *rhtXrhbABCDEF* operon to *lacZ*, a DNA fragment upstream of *rhtX* was PCR amplified using Sm_WT genomic DNA and the primers listed in Table S2. The resulting amplicon was cloned into pMP220 to give the plasmid pMPrhBIO, which was used to generate strains harboring the *rhb-lacZ* fusion (Table S1).

For β-galactosidase activity analyses, *M. xanthus* and *S. meliloti* were treated as shown in Fig. S2A. Blue color development, resulting from β-galactosidase activity in representative samples, was recorded using an Olympus (Tokyo, Japan) SZX7 dissecting microscope equipped with a DP72 digital camera and analyzed using the Olympus Cell^F software. β-Galactosidase specific activity of predator and prey in pure cultures was quantified as previously reported [49].

### Predation experiments

Two different types of assays were used to study predation, which have been termed as distance predation assays (DPAs) [16] and overlapping predation assays (OPAs) (Fig. S2). OPAs have been carried out as previously reported with modifications [54]. DPAs were used for qualitative gene expression, motility, and predation analyses. When the quantification of predation was required, OPAs were chosen. Due to the low predator cell number used in OPAs compared with that of the prey (Fig. S2B), when predator doubling times were significantly longer, a DPA approach was used to qualitatively analyze the predatory efficiency. Note that OPAs do not allow the observation of the role of motility because predation initiates immediately after spotting, whereas in DPAs, cells require ~24 h to contact.

### Quantification of *M. xanthus* and *S. meliloti* by droplet digital PCR

Genomic DNA was extracted from the cells (three drops per replicate), quantified using a NanoDrop ND-2000 spectrophotometer (NanoDrop Technologies, Wilmington, DE, USA), and digested with *Sma*I. Droplet digital PCR (ddPCR) was then conducted using the primers and probes listed in Table S2. The ddPCR reaction contained 12 μl of ddPCR Supermix for probes (no dUTP) (Bio-Rad, Hercules, CA, USA), 450 mM each of *M. xanthus* primers, 230 nM each of *S. meliloti* primers, 250 nM of *M. xanthus* probe, 150 nM of *S. meliloti* probe, 5 ng of genomic DNA from each sample tested, and molecular-grade water to a total volume of 22 μl. The PCR reaction mixture was loaded into DG32 Automated Droplet Generator Cartridges (Bio-Rad), and droplets were formed with the Automated Droplet Generator (Bio-Rad). PCR was performed in a T100 thermal cycler (Bio-Rad) under the following conditions: one denaturation hot-start cycle at 95°C for 10 min, 40 cycles of denaturation at 96°C for 30 s and annealing at 61°C for 2 min, and a final extension step at 98°C for 10 min. All the steps were performed at a ramp rate of 2°C/s. Analysis of QX200 Droplet Reader (Bio-Rad) data was performed using QX Manager Standard Edition software (Bio-Rad) to track and analyze the fluorescent drop distribution and positive detection threshold readings. Primers and probes for each organism were designed at the end portion of the replication fork to ensure that gene and chromosome copy numbers were similar (Table S2).

### Assay of *M. xanthus* motility

*M. xanthus* strains were grown and spotted as shown in Fig. S2A. The diameter of three colonies was measured every 24 h for 1 week.

### Generation time determination

Bacterial strains were grown in CTT broth to an optical density at 600 nm (OD_600_) of 1. Next, predator and prey cultures were diluted in CTT broth to an OD_600_ of 0.05. Flasks from three replicates for each condition were incubated with shaking at 30°C. Cell growth was measured spectrophotometrically at OD_600_ every 2 h, and the generation time was determined during the exponential growth phase.

### Blue chrome azurol S (CAS) agar assay for siderophore detection

Bacteria were grown and concentrated to an OD_600_ of 15. Drops of 10 μl of the bacterial suspensions were deposited onto the surface of CTT blue chrome azurol S (CAS) agar plates [55]. Plates from three replicates for each strain were incubated at 30°C, and images of representative samples were taken as mentioned above.

### Microscopy studies

#### Variable pressure scanning electron microscopy

These experiments were performed on CTT agar plates following the methodology previously described [30] using a FESEM Zeiss Supra 40Vp microscope (Jena, Germany) equipped with an energy-dispersive X-ray (EDX) microanalysis system.

#### High-resolution transmission electron microscopy

Cells from the crossing point and distal edges of *M. xanthus* and *S. meliloti* from DPAs were collected, treated, and analyzed as previously described [30], using a microscope FEI TITAN G2 (Waltham, MA, USA) equipped with a high-angle annular dark field (HAADF) type detector and an EDX microanalysis system.

#### Fluorescence microscopy

To measure ROS, 100 μl of dichloro-dihydro-fluorescein diacetate (DCFH-DA) at 1 μM was carefully added to drops of pure cultures and DPAs of predator and prey after 48 h of incubation. Fluorescein was left to act for 30 min, and images were taken using an Olympus IX71 fluorescence microscope with a DP72 digital camera. Fluorescence signals were analyzed using the Olympus Cell^F software.

## Results

### Studies on the role of iron during predation

**Figure 1:**
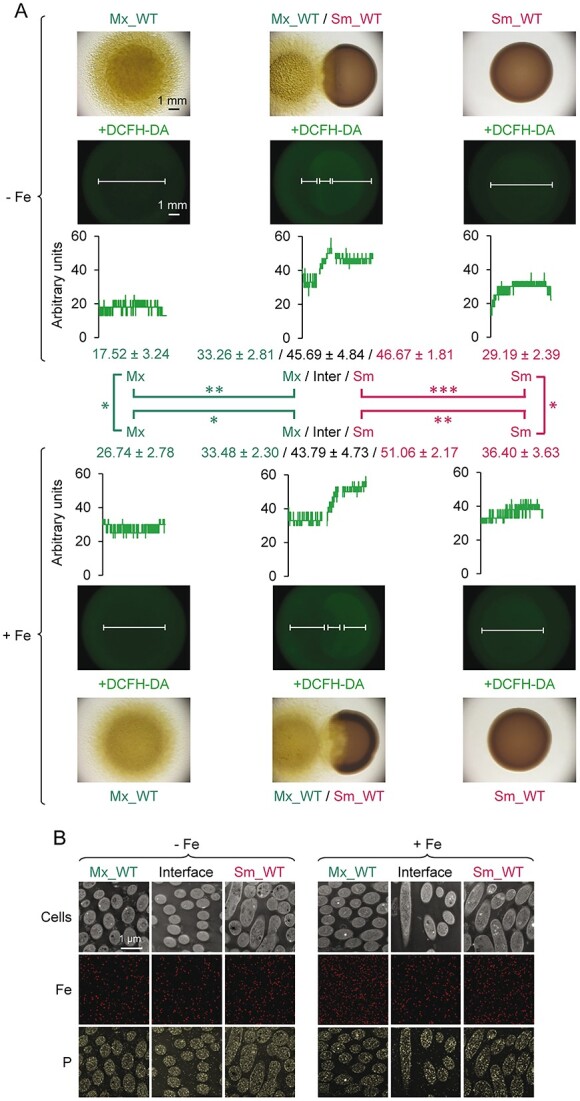
Studies on the role of iron during predation. (A) Determination of ROS accumulation in *M. xanthus* and *S. meliloti* grown in pure cultures and cocultures*.* Cells were incubated in CTT medium supplemented or not with 220 μM FeCl_3_. Pictures were taken 48 h after adding DCFH-DA under a microscope with illumination from the bottom or using a fluorescence filter. The amount of ROS was measured along the white bars in the fluorescence images. In cocultures, three measures are shown: *M. xanthus* Region (Mx), interface region (Inter), and *S. meliloti* region (Sm). Experiments were performed in triplicate, and error numbers indicate standard deviations. Two-tailed Student’s *t*-test was used to determine significant differences in ROS accumulation (^*^: *P < *0.05; ^*^^*^; *P < *0.01; ^*^^*^^*^: *P < *0.001). Comparisons between *M. xanthus* are depicted in green, and between *S. meliloti* in pink. (B) Distance predation assays of *M. xanthus* versus *S. meliloti* were performed in media without or with iron supplementation. Cells from areas where each strain was alone or at the collision point were collected at 48 h. images of the same area were obtained by HRTEM using an HAADF detector (upper pictures) or EDX microanalysis for iron (middle pictures) and phosphorus (lower pictures).

To address the role of iron during the predation of *M. xanthus* on *S. meliloti*, DPAs were performed on media with and without added iron. These results showed that *M. xanthus* penetrated more efficiently into the prey colony in media supplemented with iron (Fig. 1A), indicating that this metal favors the predatory capability of the myxobacterium. This improvement could be caused by an increase in oxidative stress in the prey generated by the accumulation of iron. To investigate this possibility, the amount of ROS in both bacteria was analyzed using DCFH-DA. The results revealed that predators and prey accumulated more ROS in cocultures than in pure cultures, although this accumulation does not appear to be related to the amount of iron present in the media (Fig. 1A). In addition, high-resolution transmission electron microscopy (HRTEM) with EDX microanalyses revealed that iron was not accumulated inside the cells, regardless of the metal levels in the medium, and no differences in iron distribution were observed between regions where strains were alone or in contact (Fig. 1B). In contrast, phosphorus detection was clearly associated with cells. These experiments ruled out iron as being responsible for the accumulation of ROS observed during predation.

**Figure 2:**
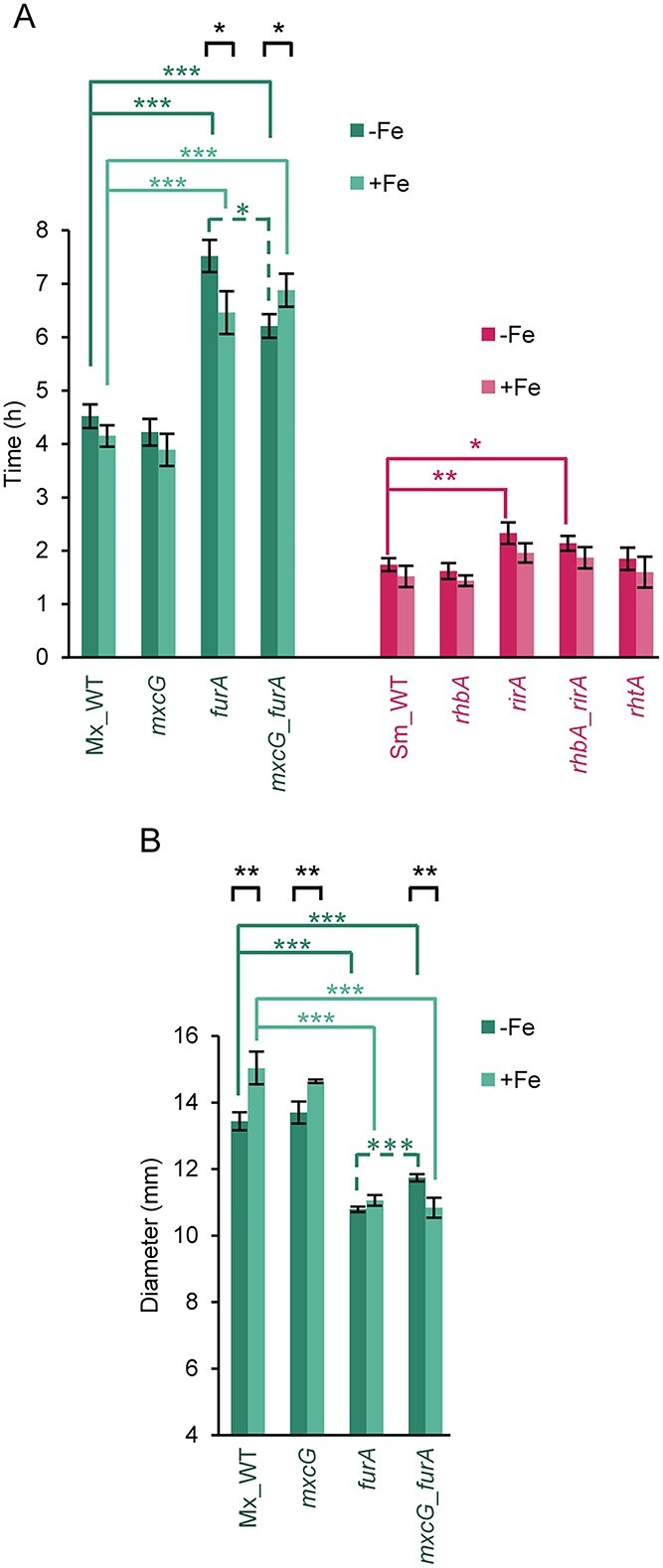
Generation time and motility of the WT and mutant strains used in this study. (A) Doubling time of myxobacterial (green bars) and rhizobial (pink bars) strains grown in CTT medium with 220 μM FeCl_3_ or without iron supplementation. (B) Expansion of *M. xanthus* strains in CTT medium with and without iron addition. Diameter of the colonies was measured after 48 h of incubation. Experiments were performed in triplicate, and error bars indicate standard deviations. Two-tailed Student’s *t*-test was used to determine significant differences (^*^: *P < *0.05; ^*^^*^: *P < *0.01; ^*^^*^^*^: *P < *0.001), which are shown following this code: Comparisons of the WT strains with mutants (green for *M. xanthus* and pink for *S. meliloti*) grown under the same conditions are depicted with continuous lines; comparisons of the *M. xanthus mxcG_furA* mutant versus the *furA* mutant grown under the same conditions are depicted with green dashed lines; and comparisons between the same strain grown in media with or without iron supplementation are depicted as black continuous lines.

To further investigate why iron improves predation, generation times of predator and prey wild-type (WT) strains were compared in media supplemented or not with the metal, and the results showed that iron does not significantly modify these values (Fig. 2A). Furthermore, because *M. xanthus* motility is required for proper predation [16], the expansion rate of the Mx_WT colony was also measured, revealing that the diameter of the colony significantly increased in media supplemented with iron (Fig. 2B). To rule out the possibility that this increase could be the result of growth, the net radial expansion of the colony over time, which is equal to the slope of the straight line obtained when growth rate is plotted against motility, was determined [56]. The results showed that the motility of Mx_WT increased by 12% in media supplemented with iron. Altogether, these experiments indicate that oxidative stress increases during predatory interactions and that iron does not appear to be responsible for ROS accumulation. However, iron improves predation, which correlates with a higher motility rate in *M. xanthus*.

### Iron and predator and prey siderophores accumulate at the predatory interface in media without iron supplementation

**Figure 3:**
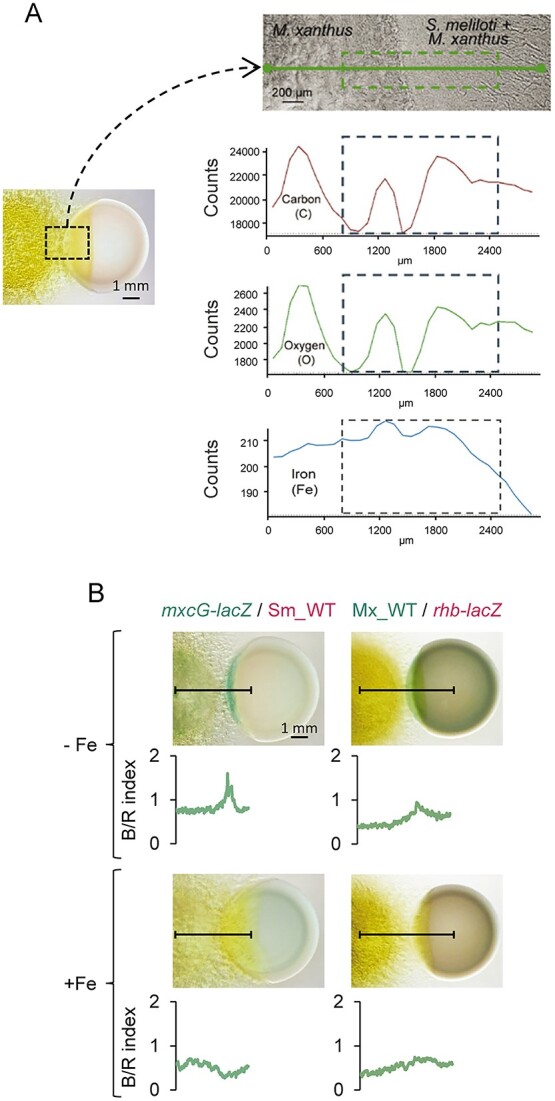
Iron and siderophores accumulate at the predator–prey interface. (A) Determination of carbon, oxygen, and iron at the interface predator–prey by VPSEM with EDX microanalysis. Only the graph corresponding to iron is new in this manuscript. The rest of the images were obtained from our previous work [30], with permission. (B) Expression of genes involved in myxochelin (left) and Rz1021 (right) biosynthesis during the predation of *M. xanthus* on *S. meliloti.* Strains harboring the indicated *lacZ* fusions were assayed against the WT strain of the other bacterium in CTT medium containing X-gal, with or without iron supplementation. Pictures were taken after 48 h of incubation with illumination from the top (left pictures) or from the bottom (right pictures). Colors were scanned along the lines drawn in the pictures. To eliminate background from white light, the values shown are the ratio between blue and red (B/R index).

To focus on the role of competition for iron during predation, the amount of iron at the interface where both bacteria collided on CTT medium was determined using variable pressure scanning electron microscopy (VPSEM) coupled with EDX. Scan microanalyses using this technique revealed that whereas carbon and oxygen (indicative of cell biomass) exhibited similar profiles, roughly corresponding to the accumulation of cells in the area, iron levels especially increased in those regions where both bacteria contact, following a pattern different from that described by the biomass (Fig. 3A). Because an increase in the amount of metal inside the cells was not detected (Fig. 1B), these results suggest that iron remains extracellularly in the predatory interface.

Previous findings revealed that the *M. xanthus* and *S. meliloti* siderophore biosynthesis genes (Fig. S3) are upregulated during predation (Fig. S4) [40, 41]. To monitor this upregulation in *M. xanthus*, an *mxcG-lacZ* fusion was constructed*.* The *mxcG* gene encodes a nonribosomal peptide synthetase involved in the biosynthesis of myxochelins [57, 58], which are siderophores produced by *M. xanthus* (Fig. S3A). The results showed that the predator increased the expression of myxochelin biosynthesis genes when encountering the prey in a medium not supplemented with iron (Fig. 3B). Similarly, an *S. meliloti* strain harboring an *rhb-lacZ* fusion was constructed. *rhb* genes are involved in Rz1021 biosynthesis (Fig. S3B), the siderophore produced by *S. meliloti* Rm1021 [59]. DPAs confirmed that in medium nonsupplemented with iron, the prey also upregulated the expression of siderophore biosynthesis genes (Fig. 3B). As expected, the addition of iron repressed the expression of both myxochelin and Rz1021 biosynthesis genes (Fig. 3B). Altogether, these data indicate that competition for iron occurs during predation, which might be decisive in the outcome of the interaction. To address this question, efforts were focused on studying the role of siderophores during predation.

### Predator siderophores improve predation

**Figure 4:**
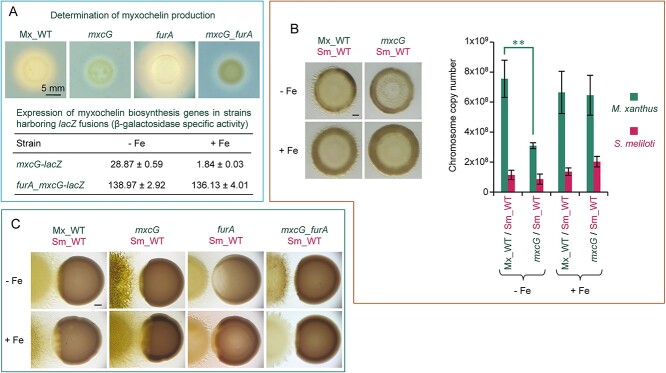
Influence of myxochelin production on the predatory capability of *M. xanthus*. (A) Analysis of the biosynthesis of myxochelins in several strains of *M. xanthus*. In the upper pictures, the Mx_WT strain and *mxcG*, *furA*, and *mxcG_furA* mutants were cultured on blue CAS agar for 72 h. The yellow halo around the colony originates from the accumulation of siderophores. Data in the lower part of the panel show the expression levels of myxochelin biosynthesis genes in the Mx_WT strain (*mxcG-lacZ*) and *furA* mutant (*furA_mxcG-lacZ*). Cells harboring fusions between myxochelin biosynthesis genes and *lacZ* were incubated on CTT medium with or without iron supplementation for 24 h. Three samples were then collected, and β-galactosidase specific activity was quantified using *o*-nitrophenyl galactopyranoside as a substrate. Specific activity is expressed as nmol of *o*-nitrophenol produced per min and mg of protein. (B) Predatory capability of the Mx_WT strain and the *mxcG* mutant, which does not produce myxochelins (*mxcG* deletion), on Sm_WT in media with or without iron addition. Assays were performed for 72 h in semiquantitative overlapping predation assays (left pictures) and quantified by ddPCR (right graph). ddPCR experiments were performed in triplicate, and error bars indicate standard deviations. Significant differences between WT strains and mutants grown under the same conditions were determined using a two-tailed Student’s *t*-test (^*^^*^: *P < *0.01) and are depicted with continuous lines. (C) Qualitative analysis of the predatory behavior of a mutant that overproduced myxochelins (*furA* gene is deleted) in CTT media with or without iron addition, compared with the Mx_WT strain and the *mxcG* and *mxcG_furA* mutants. All images in this figure were taken at 72 h under a dissecting microscope with illumination from the bottom. Bars represent 1 mm unless otherwise stated.

To investigate the role of predator siderophores, a myxochelin-deficient mutant (*mxcG*) was obtained (Fig. 4A). OPAs were performed to quantify the number of cells after predator–prey interaction. When the predatory activity of the *mxcG* mutant was compared with that of Mx_WT, it was observed that, without iron addition, the number of rhizobial chromosomes estimated by ddPCR was similar when exposed to the Mx_WT strain and the *mxcG* mutant, whereas the number of *M. xanthus* chromosomes detected in the *mxcG* mutant was approximately half of that of the Mx_WT strain (Fig. 4B). Although the *mxcG* mutant exhibits a doubling time and an expansion rate similar to those of the Mx_WT strain in pure cultures (Fig. 2), the data obtained during predation suggest that the prey may interfere with the growth of this mutant. In fact, the expression of genes involved in Rz1021 biosynthesis (detected by using an *rhb-lacZ* strain) was high even when *S. meliloti* was confronted with the nonsiderophore producer *mxcG* mutant (Fig. S5). In contrast, when iron was added to the medium and siderophore biosynthesis genes were not expressed in any strain (Fig. 4A), the number of predator chromosomes (Mx_WT and *mxcG* mutant) after the interaction was similar (Fig. 4B).

To further investigate the role of myxochelins in predation, we decided to construct a mutant that overproduced these siderophores even in media supplemented with iron. Therefore, it was first necessary to identify the repressor that regulates the expression of myxochelin biosynthesis in *M. xanthus*. An analysis of the genome revealed that the gene MXAN_3702 encodes a protein with similarities to Fur family members. To investigate the function of this protein, a strain harboring a deletion in this gene was generated. This mutant overproduced siderophores and expressed myxochelin biosynthesis genes at high levels, with and without iron supplementation (Fig. 4A), demonstrating that this repressor, termed FurA, is the master regulator of siderophore production in *M. xanthus*. The *furA* mutant exhibited a longer doubling time and a lower expansion rate than the Mx_WT (Fig. 2). Therefore, when the predatory capability of the *furA* mutant was assayed in OPAs, it was found that it exhibited a diminished ability to grow and kill the prey (Fig. S6). This result might be explained by the fact that, in OPAs, the number of predator cells is ≈100 times lower than that of the prey (Fig. S2B), which are unable to efficiently grow in the presence of a robust Sm_WT with a shorter doubling time (Fig. 2). To overcome this difficulty and discern the role of myxochelins in predation, DPAs were performed using the *furA* mutant as a predator. In this case, without iron addition, the area of the prey colony close to the predator was clearer, even before the predator had reached it, compared with the region far from the mutant (Fig. 4C). This observation can be explained by considering that the mutant overproduces myxochelins (Fig. 4A), which can diffuse and reach the prey colony in advance, depleting iron for the prey and inhibiting its growth. In contrast, in CTT with iron, in which this metal is not limiting to prey, the behavior of the *furA* mutant and Mx_WT strain appeared to be similar (Fig. 4C). Hence, because the *furA* mutant, in addition to overproducing myxochelins, exhibits other phenotypic defects, a double mutant *mxcG_furA* (in which myxochelin production was abolished) was generated and analyzed to confirm the role of siderophores in predation (Fig. 4A). This mutant retained defects in growth rate and motility (Fig. 2). The results in DPAs revealed that this double mutant does not inhibit growth of the prey before reaching it as the *furA* mutant does (Fig. 4C), indicating that this inhibition of growth observed in the prey by the *furA* mutant is caused by myxochelins. Therefore, the results obtained with *M. xanthus* mutants (nonproducers and overproducers of myxochelins) indicate that predator siderophores decrease iron availability for prey and facilitate predation.

### Prey siderophores contribute to defense against predator attack

**Figure 5:**
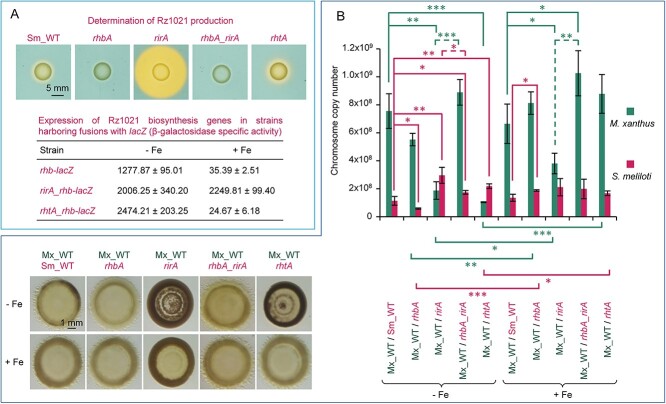
Influence of Rz1021 production on the defensive capability of *S. meliloti* against predation. (A) Analysis of Rz1021 production in the rhizobial strains used in this study. In the upper images, rhizobial strains were cultured on blue CAS agar for 48 h. The yellow halo around the colony results from the accumulation of siderophores. Data in the lower part of the panel show the expression levels of Rz1021 biosynthesis genes in the Sm_WT strain (*rhb-lacZ*) and *rirA* and *rhtA* mutants (*rirA_rhb-lacZ* and *rhtA_rhb-lacZ*, respectively). Cells harboring fusions between Rz1021 biosynthesis genes and *lacZ* were incubated in CTT medium with or without iron supplementation for 24 h. Three samples were then collected, and β-galactosidase specific activity was quantified using *o*-nitrophenyl galactopyranoside as a substrate. Specific activity is expressed as nmol of *o*-nitrophenol produced per min and mg of protein. (B) Predatory behavior of the Mx_WT strain against the Sm_WT and mutant strains that do not produce (*rhbA* and *rhbA_rirA* mutants) or overproduce (*rhtA* and *rirA* mutants) Rz1021 was determined at 72 h of incubation by semiquantitative analysis of the cocultures with or without iron supplementation (left pictures). Pictures were taken with illumination from the bottom. Quantification was performed by ddPCR after 72 h of interaction (right graph). ddPCR experiments were performed in triplicate, and error bars indicate standard deviations. Two-tailed Student’s *t*-test was used to determine significant differences (^*^: *P < *0.05; ^*^^*^: *P < *0.01; ^*^^*^^*^: *P < *0.001), which are shown following this code: Comparisons of the WT strains with mutants (green for *M. xanthus* and pink for *S. meliloti*) grown under the same conditions are depicted with continuous lines (top); comparisons of the same strain in the same interaction grown with and without iron are also depicted with continuous lines (bottom); comparisons of Mx_WT versus *rirA* and *rhbA_rirA* mutants grown under the same conditions are depicted with dashed lines (green for *M. xanthus* and pink for *S. meliloti*).

The role of prey siderophores during predation was also analyzed by comparing the behavior of Sm_WT with that of mutants altered in the production and uptake of Rz1021 (Fig. 5A) [60]. Predation experiments performed with the nonRz1021 producer *rhbA* mutant revealed reduced survival of the prey to predatory attack in iron-limited media but not in media supplemented with the metal (Fig. 5B). As this mutant does not exhibit a lower growth rate than the Sm_WT strain (Fig. 2A), the decrease of survival of the *rhbA* mutant indicates that iron acquisition facilitated by Rz1021 contributes to prey resistance to predatory attack.

To investigate the role of siderophores in prey resistance, a strain that overproduced Rz1021 was generated by deletion of the *rirA* gene, which encodes the repressor of Rz1021 biosynthesis genes [61]. The *rirA* mutant overproduced siderophores, expressed Rz1021 biosynthesis genes regardless of the iron levels in the medium (Fig. 5A), and exhibited a longer doubling time than Sm_WT (Fig. 2A). In media without iron supplementation, the *rirA* mutant resisted predation (Fig. 5B). Moreover, the number of Mx_WT chromosomes when confronted with the *rirA* mutant was lower that when exposed to Sm_WT, even in media supplemented with iron (Fig. 5B), indicating that *M. xanthus* has difficulties growing when Rz1021 is overproduced. Note that *M. xanthus* upregulated myxochelin biosynthesis genes during interaction with the *rirA* mutant, even in CTT supplemented with iron (Fig. S7). Hence, because the *rirA* mutant upregulates the expression of several other genes in addition to those involved in the biosynthesis of siderophores [61], a double mutant *rhbA_rirA* was constructed (in which siderophore production was abolished) to confirm the role of Rz1021 in predation (Fig. 5A). The results showed that this double mutant was more sensitive to predation than the *rirA* mutant (Fig. 5B), confirming that the resistance to predation of the *rirA* mutant is caused by Rz1021 overproduction.

To corroborate the role of Rz1021, the gene coding for the TBDT RhtA, which is required for transporting Fe-Rz1021 through the outer membrane [59], was inactivated. The *rhtA* mutant overexpressed Rz1021 biosynthesis genes only when iron was not added (Fig. 5A) because it was unable to take up ferrisiderophores. In contrast to the *rirA* mutant, the doubling time of the *rhtA* mutant was similar to that of the Sm_WT (Fig. 2A). Predation assays revealed that the *rhtA* mutant was more resistant to Mx_WT predation than the Sm_WT in media not supplemented with iron (Fig. 5B). However, when iron was added to the media and Rz1021 biosynthesis genes were not expressed (Fig. 5A), survival levels of the *rhtA* mutant and the Sm_WT were similar (Fig. 5B), confirming that Rz1021 protects *S. meliloti* from predation by depleting the iron supply from the predator.

### Predatory capability of predator mutants against prey mutants

**Figure 6:**
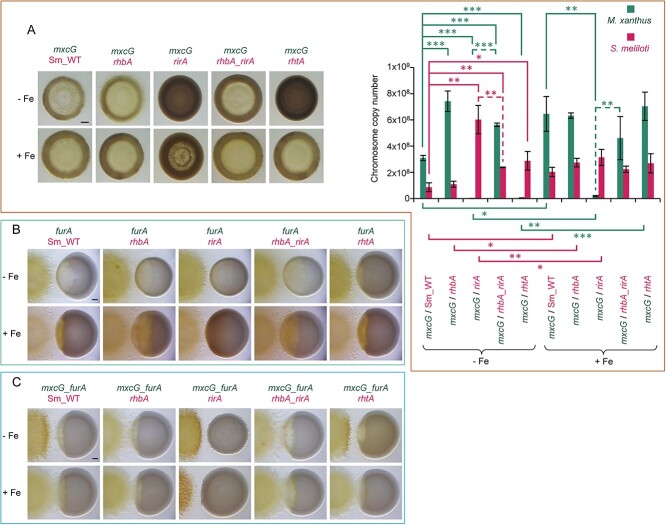
Analysis of the predatory behavior of mutants of *M. xanthus* that do not produce or overproduce myxochelins when they are cocultured with mutants of *S. meliloti* that do not produce or overproduce Rz1021 in CTT media with or without iron supplementation. (A) Cocultures of the *M. xanthus mxcG* mutant with the *S. meliloti* Sm_WT*, rhbA*, *rirA*, *rhbA_rirA*, or *rhtA* strains were monitored after 72 h of interaction by semiquantitative analyses in media with or without iron addition (left pictures) and quantified by ddPCR (right graph). ddPCR experiments were performed in triplicate, and error bars indicate standard deviations. Significant differences were determined using a two-tailed Student’s *t*-test (^*^: *P < *0.05; ^*^^*^: *P < *0.01; ^*^^*^^*^: *P < *0.001). Comparisons of the coculture data of the *M. xanthus mxcG* mutant (green lines) against Sm_WT with those obtained against all the rhizobial mutants (pink lines) grown under the same conditions are depicted as continuous lines (top); comparisons of the same strain in the same interaction grown with and without iron are also depicted with continuous lines (bottom); comparisons of the data with the *mxcG* mutant versus *rirA* and *rhbA_rirA* mutants grown under the same conditions are depicted with dashed lines (green for *M. xanthus* and pink for *S. meliloti*). (B) Predatory behavior of the *furA* mutant of *M. xanthus* in distance predation assays in media with or without iron. (C) Predatory behavior of the *mxcG*_*furA* mutant of *M. xanthus* in distance predation assays. The pictures in the three panels were taken at 72 h under a dissecting microscope with illumination from the bottom. Bars in the pictures represent 1 mm.

To further test the role of siderophores, predator mutants were analyzed for their predatory behavior against prey mutants. The results revealed that the *mxcG* mutant, a nonproducer of myxochelins, could not grow properly when confronted with rhizobia that overproduced Rz1021 (*rirA* and *rhtA* mutants in media without added iron, and only *rirA* mutant in media with iron supplementation) (Fig. 6A). When *mxcG* was assayed against mutants nonproducers of Rz1021 (*rhbA* and *rhbA_rirA*), the number of predator chromosomes obtained (Fig. 6A) was similar to that observed in the Mx_WT (Fig. 5A).

When predation was carried out by the *furA* mutant, which always overexpressed myxochelin biosynthesis genes (Fig. 4A), qualitative analyses in DPAs revealed that, in media not supplemented with iron, this mutant penetrated the rhizobial colonies of nonproducer mutants of Rz1021 (*rhbA* and *rhbA_rirA* mutants) at a similar rate than in colonies of the Sm_WT (Fig. 6B). However, under the same conditions, the *furA* mutant could not efficiently penetrate the *rhtA* and *rirA* mutant colonies (Fig. 6B). These results correlate with the amount of Rz1021 produced by these two rhizobial mutants (Fig. 5A). When the same mutants were assayed in iron-enriched media, the *furA* mutant penetrated very efficiently the colonies of *rhbA*, *rhbA_rirA*, and *rhtA* mutants*,* but not in the *rirA* mutant colony (Fig. 6B), corresponding to the fact that *rhbA*, *rhbA_rirA*, and *rhtA* mutants do not synthesize Rz1021 under these conditions, whereas the *rirA* mutant does (Fig. 5A). The efficiency of the *furA* mutant to penetrate the rhizobial colonies is the result of myxochelin overproduction, as indicated by the reduced ability to penetrate the rhizobial colonies exhibited by the myxochelin-deficient *mxcG_furA* double mutant (Fig. 6C). These data confirm that competition for iron is a central battlefield between predator and prey and that siderophores alter the outcome of predator–prey interactions.

## Discussion

Myxobacterial predation is a multifactorial process in which cells use a vast arsenal to hunt, kill, lyse, and consume prey [62], which varies from one prey to another [20, 36, 40, 63, 64]. Within this arsenal, metals are emerging as novel weapons to kill prey [30, 36, 39]. In this study, we focused on the role of iron and siderophores during the predatory interaction of *M. xanthus* with *S. meliloti*.

Studies have revealed that intoxication with iron is not a major mechanism used by the predator because the metal is not accumulated inside the cells and the accumulation of ROS in cells during predation is similar regardless of the amount of metal included in the media (Fig. 1). Moreover, transcriptomic studies during the predation of *M. xanthus* on *S. meliloti* have shown that genes involved in the detoxification of ROS are not upregulated in the prey, except for superoxide dismutase [41]. Similarly, no gene upregulated during predation in the predator appears to play a role in iron intoxication of the prey [40]. However, iron improves penetration of the predator into the prey colony, because the expansion rate of the predator increases with iron, which undoubtedly favors reaching the prey and establishing close contact with the cells. Nevertheless, it cannot be ruled out that several other mechanisms used by the predator may be more efficient in iron-enriched media.

In contrast to intoxication with iron, competition for this metal appears to be decisive in the outcome of the predator–prey interaction. Data obtained in this work with mutants that do not produce or overproduce siderophores demonstrate that the competitor that is able to deplete the iron supply for the rival will prevail. Other studies also point in this direction. For instance, an *M. xanthus* mutant in MXAN_6911, which is a putative ferrimyxochelin transporter, exhibits less efficient predation on *P. aeruginosa* [39]. However, this mutation has no effect on intracellular iron levels or siderophore synthesis, probably because there are two types of myxochelins, A and B, which could be transported by different TBDTs. Three genes that encode putative TBDTs (MXAN_1316, MXAN_5023, and MXAN_6911) are upregulated during predation on *S. meliloti* (Fig. S4A), with the upstream regions of MXAN_5023 and MXAN_6911 exhibiting a Fur box [40]. In addition, a previous study showed that mutants in components of the ABC transporter that introduces ferrimyxochelins into the *M. xanthus* cytoplasm are defective in predation [39]. This study also generated a mutant that produced fewer siderophores than the Mx_WT strain, which was also defective in predation [39]. However, the pathway involved in myxochelin A and B biosynthesis from chorismate, depicted in Fig. S3A, has been well established [57, 58], and the gene that these authors mutated (MXAN_3618), although encoding a nonribosomal peptide synthetase, has not been reported to be involved in this process. Therefore, it cannot be ruled out that other processes, in addition to myxochelin biosynthesis, can be impaired in this mutant, which may also be involved in predation.

Several transcriptomes using *M. xanthus* as a predator against diverse prey, such as *S. coelicolor* [36], *E. coli*, and *M. luteus* [42], have been published, and in all of them, siderophore biosynthesis genes were upregulated in both predator and prey. Moreover, using another myxobacterium as a predator (*C. ferrugineus*) against *P. putida*, the same result was obtained [38], which indicates that competition for iron may be a general mechanism that participates in myxobacterial predation. These transcriptomes have also revealed that predation is a dynamic process, where the profiles of predator and prey genes differentially expressed at earlier and later times of the interaction are not identical, denoting that each bacterium adapts to the response of the other. In the case of the predation of *M. xanthus* on *S. coelicolor*, the depletion of iron triggers the biosynthesis of the antibiotic actinorhodin in the prey [36], indicating that competition for this metal functions as a first line of attack-defense during the interaction.

Two metals have been reported to be used by *M. xanthus* during its predatory interaction with *S. meliloti*: copper and iron. Copper appears to be used to kill prey by oxidative stress because the metal accumulates inside the cells, the predator upregulates the expression of genes involved in copper detoxification, and the prey responds by synthesizing melanin to protect from oxidative stress [30]. In the case of iron, competition for the metal appears to be more relevant than intoxication.

At pH 7, iron has a solubility of 1.4 × 10^−9^ M [65], which makes it very inaccessible to organisms. Moreover, many metalloenzymes require iron as a cofactor [66, 67]. To overcome this problem, the production of siderophores is the main mechanism used by bacteria for scavenging the limited amount of iron in the environment [30], and ample information is available about the chemical nature of these iron chelators, the biochemical pathways responsible for their biosynthesis, the mechanisms involved in the uptake of the ferrisiderophores, and the regulation of the genes involved in the entire process [32–35]. There are also abundant studies on the antagonism among bacteria in their habitats to compete for this metal and between pathogens and their hosts [68–73]. However, not much is known about the ecological impact of competition for iron through the production of siderophores [74]. In particular, the consequences of competition for this metal in predator–prey interactions have not been thoroughly investigated, although siderophores can modify soil properties by iron sequestration [75–77]. Data obtained in this work indicate that the production of siderophores by microorganisms and fluctuations in iron concentration in the habitats are expected to affect the outcome of the predator–prey interaction, determining which population will predominate. In fact, prey that overproduce Rz1021 (*rirA* and *rhtA* mutants) impose over the predator (Figs 5B and 6A), reversing the outcome of predation. In the case of the interaction between *M. xanthus* and *Pseudomonas fluorescens*, temperature has been reported to determine the direction of predation, also reversing predator and prey identities [78]. Considering that myxobacteria are major micropredators in soils and are in part responsible for the diversity of microbial communities, these results may be of great interest to the environment and agriculture.

## Supplementary Material

supplementary_material_wrae077

## Data Availability

The authors declare that materials described in the manuscript, including all relevant raw data, will be freely available to any researcher wishing to use them for noncommercial purposes, without breaching participant confidentiality.
