# Supplementary material for: Siderophores and competition for iron govern myxobacterial predation dynamics
Source: ISME J. 2024 May 2;18(1):wrae077. doi: 10.1093/ismejo/wrae077 (PMC11388931; doi:10.1093/ismejo/wrae077)
Supplement: supplementary_material_wrae077 [file supplementary_material_wrae077.zip › Figure S1.pdf]

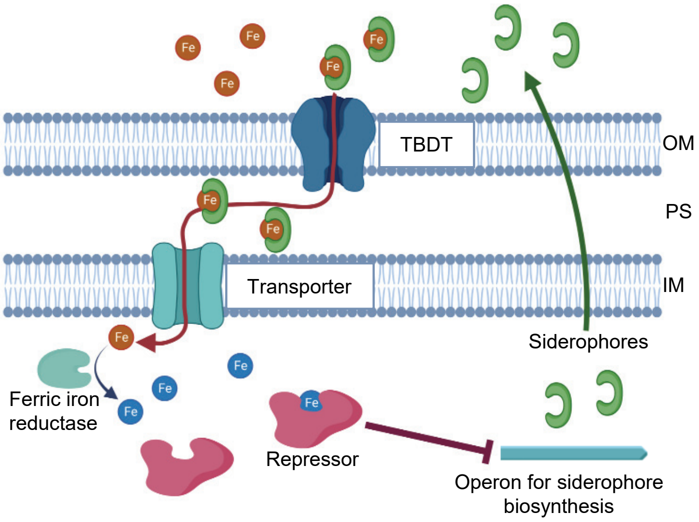

Figure S1. General scheme of the biosynthesis of siderophores and uptake of ferrisiderophores in Gram-negative bacteria. Genes involved in the biosynthesis of siderophores are under the control of a repressor that is active under iron-replete conditions. In the absence of this metal, gene repression is relieved and siderophores are synthesized and exported to the exterior, where they bind ferric ions with high affinity (brown balls). Ferrisiderophores are recognized by an outer membrane receptor (TBDT), which is energized by a TonB system to introduce them into the periplasm. They are then transported through the inner membrane by transporters (usually an ABC transporter) to the cytoplasm, where the ferric ions are reduced to ferrous ions (blue balls) by a ferric-iron reductase. OM, outer membrane; PS, periplasmic space; IM, inner membrane.
