# Supplementary material for: Siderophores and competition for iron govern myxobacterial predation dynamics
Source: ISME J. 2024 May 2;18(1):wrae077. doi: 10.1093/ismejo/wrae077 (PMC11388931; doi:10.1093/ismejo/wrae077)
Supplement: supplementary_material_wrae077 [file supplementary_material_wrae077.zip › Figure S2.pdf]

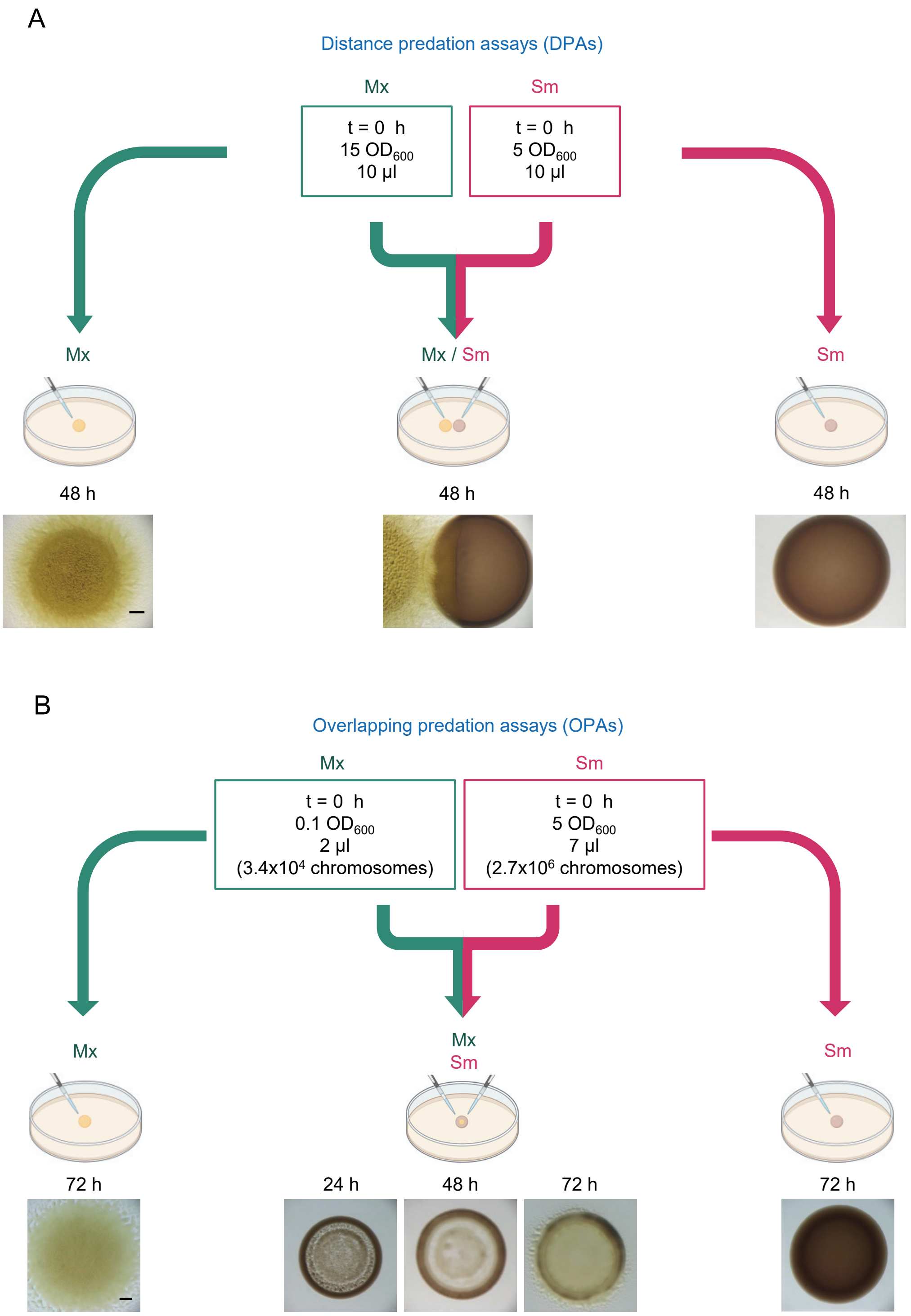

Figure S2. Methodology used to study the interaction between *M. xanthus* and *S. meliloti*. **A.** Distance predation assays (DPAs). Cells were grown to an OD_{600} of 1, centrifuged, and concentrated in TM buffer (10 mM Tris-HCl [pH 7.6], 1 mM $MgSO_4$) to OD_{600} of 15 for *M. xanthus* and 5 for *S. meliloti* strains. Drops of 10 µl of the *S. meliloti* suspensions were deposited onto the surface of CTT agar plates and allowed to dry. Next, 10 µl-drops of *M. xanthus* suspensions were spotted near the *S. meliloti* spots (no more than 1 mm separation between spots). Plates were incubated at 30°C. **B.** Overlapping predation assays (OPAs). Predator and prey were cultured as mentioned in panel A. Then, *M. xanthus* cultures were diluted in TM buffer to OD_{600} of 0.1, whereas *S. meliloti* cultures were centrifuged and concentrated in TM buffer to OD_{600} of 5. Drops of 7 µl of the *S. meliloti* suspensions were deposited onto the surface of CTT agar plates and allowed to dry. Next, 2 µl-drops of *M. xanthus* suspensions were spotted on top of the *S. meliloti* spots. Plates were incubated at 30°C. The number of chromosomes at the start of the experiment was determined by ddPCR. Bars in both panels represent 1 mm.
