# Supplementary material for: Siderophores and competition for iron govern myxobacterial predation dynamics
Source: ISME J. 2024 May 2;18(1):wrae077. doi: 10.1093/ismejo/wrae077 (PMC11388931; doi:10.1093/ismejo/wrae077)
Supplement: supplementary_material_wrae077 [file supplementary_material_wrae077.zip › Figure S3.pdf]

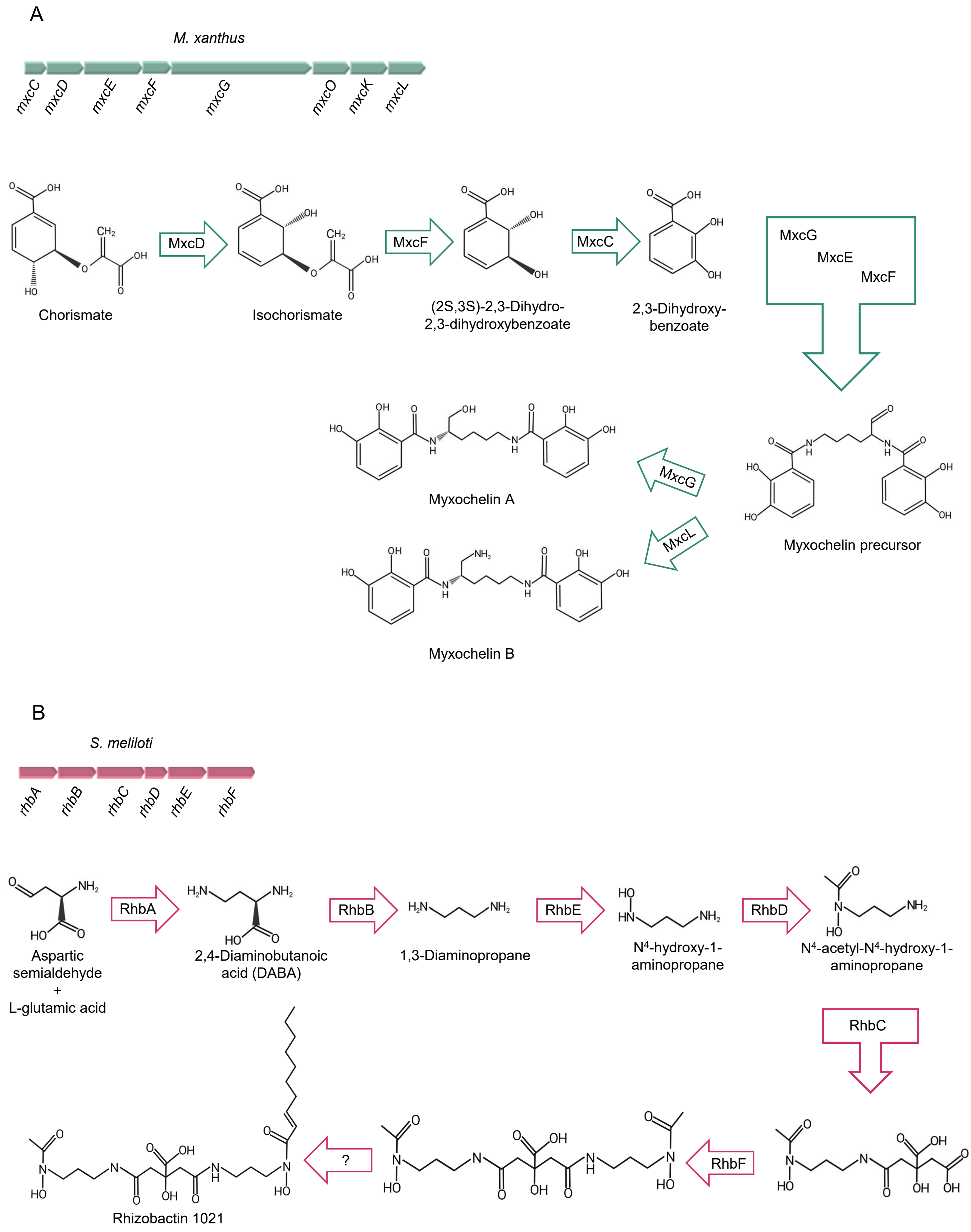


**Figure S3.** Biosynthesis of myxochelins in *M. xanthus* (**A**) and Rz1021 in *S. meliloti* (**B**). In both panels, the operon encoding the enzymes involved in siderophore biosynthesis is depicted in the upper part. In the bottom part, pathways for siderophore biosynthesis are illustrated.
