# Supplementary material for: Siderophores and competition for iron govern myxobacterial predation dynamics
Source: ISME J. 2024 May 2;18(1):wrae077. doi: 10.1093/ismejo/wrae077 (PMC11388931; doi:10.1093/ismejo/wrae077)
Supplement: supplementary_material_wrae077 [file supplementary_material_wrae077.zip › Figure S4.pdf]

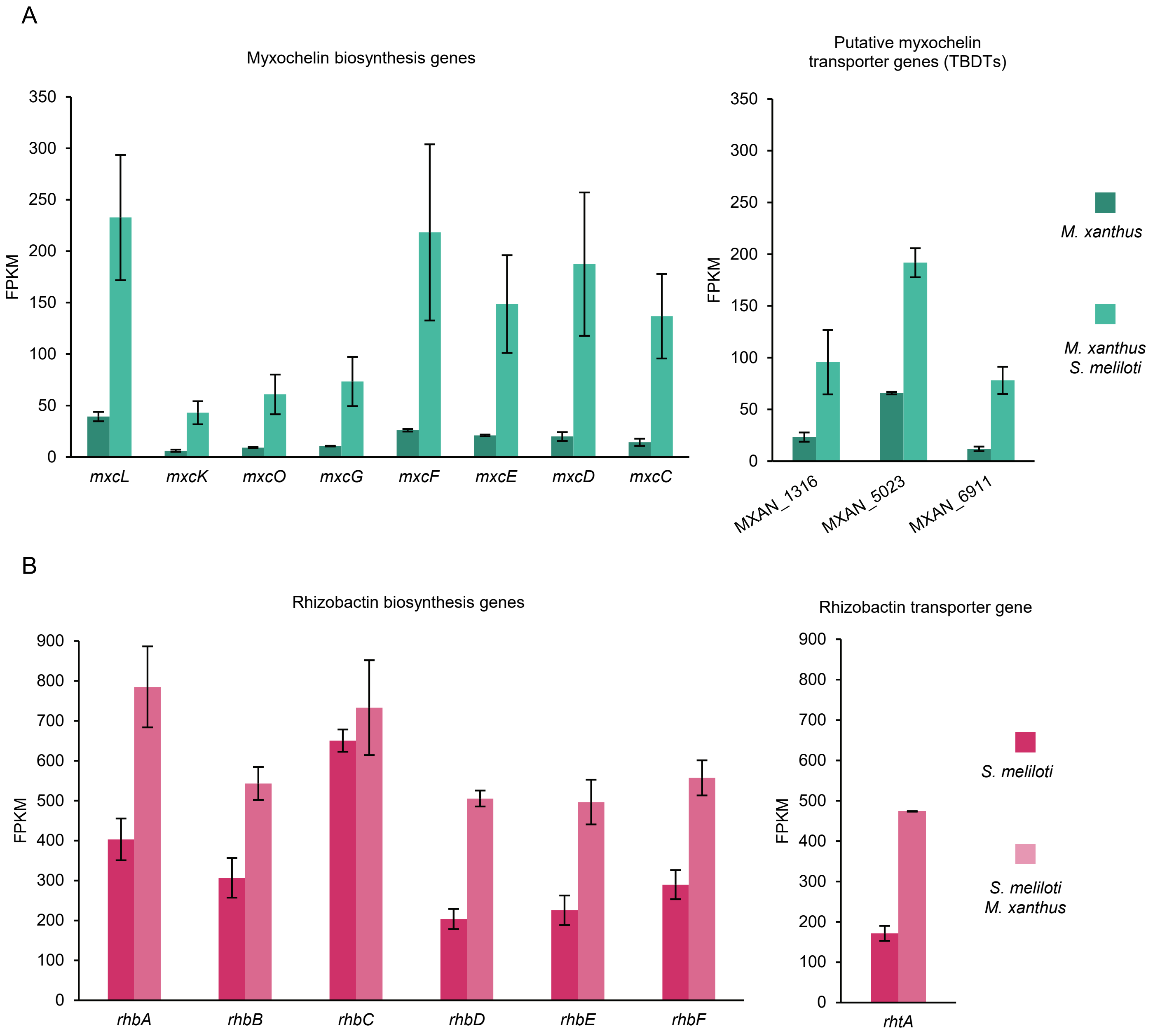


**Figure S4.** Expression of genes involved in siderophore biosynthesis (left) and ferrisiderophore uptake (right) in *M. xanthus* (**A**) and *S. meliloti* (**B**) cells grown in pure cultures (dark bars) or cocultures (light bars) for 6 h. Transcriptomic data, expressed as FPKMs (fragments per kilobase of transcript per million fragments mapped), for *M. xanthus* were obtained from Pérez et al. (2022), and for *S. meliloti* from Soto et al. (2023). Error bars indicate standard deviations.

References

Pérez J, Contreras-Moreno FJ, Muñoz-Dorado J, Moraleda-Muñoz A. Development versus predation: Transcriptomic changes during the lifecycle of *Myxococcus xanthus*. *Front Microbiol*. 2022; 13:1004476.

Soto MJ, Pérez J, Muñoz-Dorado J, Contreras-Moreno FJ, Moraleda-Muñoz A. Transcriptomic response of *Sinorhizobium meliloti* to the predatory attack of *Myxococcus xanthus*. *Front Microbiol*. 2023; 14:1213659.
