# Supplementary material for: Siderophores and competition for iron govern myxobacterial predation dynamics
Source: ISME J. 2024 May 2;18(1):wrae077. doi: 10.1093/ismejo/wrae077 (PMC11388931; doi:10.1093/ismejo/wrae077)
Supplement: supplementary_material_wrae077 [file supplementary_material_wrae077.zip › Figure S5.pdf]

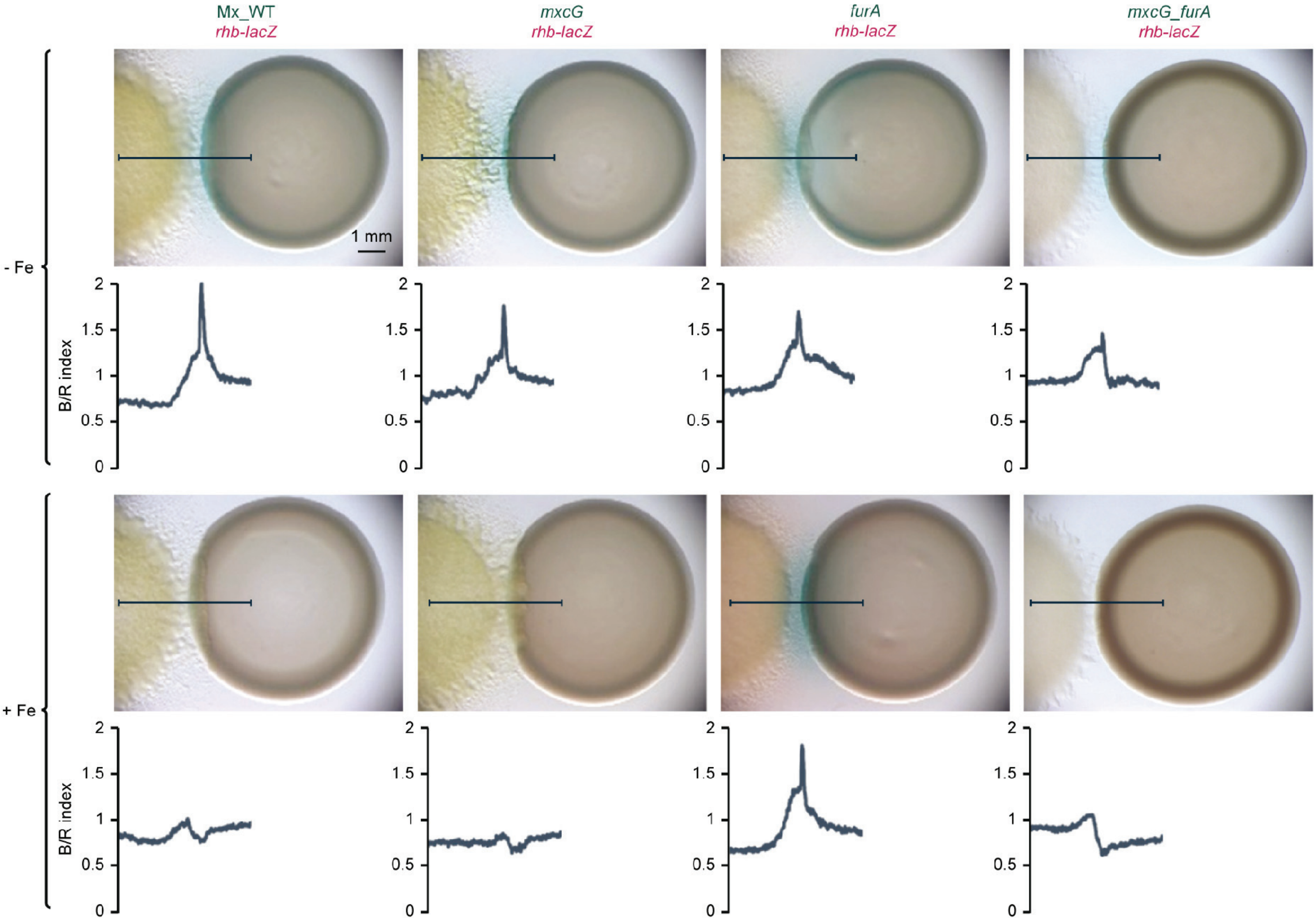

Figure S5. Expression of genes involved in Rz1021 biosynthesis when *S. meliloti* is exposed to different *M. xanthus* strains. An *S. meliloti* strain harboring a fusion between the promoter region of the *rhb* gene-containing operon and *lacZ* (*rhb-lacZ*) was assayed against the Mx_WT strain and the *mxcG*, *furA*, and *mxcG_furA* mutants. Cells were incubated in CTT medium with or without iron supplementation and X-gal to monitor β-galactosidase activity. Pictures were taken at 72 h under a dissecting microscope with illumination from the bottom. Colors were scanned along the lines drawn in the pictures. To eliminate background from white light, the values shown are the ratio between blue and red (B/R index).
