# Supplementary material for: Siderophores and competition for iron govern myxobacterial predation dynamics
Source: ISME J. 2024 May 2;18(1):wrae077. doi: 10.1093/ismejo/wrae077 (PMC11388931; doi:10.1093/ismejo/wrae077)
Supplement: supplementary_material_wrae077 [file supplementary_material_wrae077.zip › Figure S6.pdf]

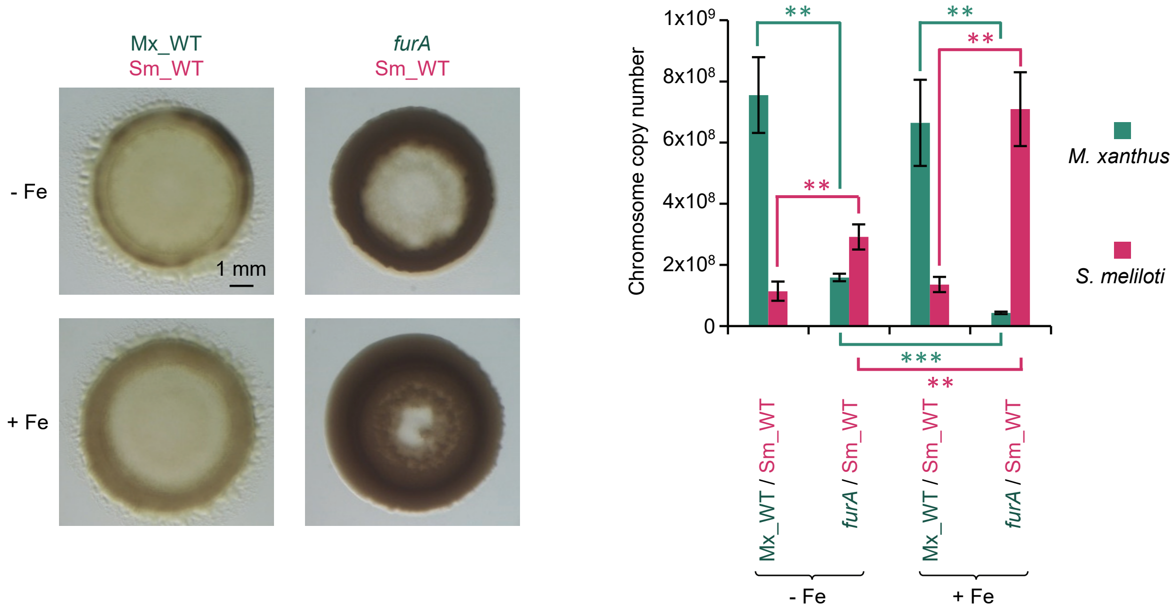

Figure S6. Predatory capability of the *M. xanthus furA* mutant (overproducer of siderophores) determined after 72 h of interaction with the WT strain of *S. meliloti* in media with and without iron added by semiquantitative analyses (left pictures) and quantified by ddPCR (right graph). ddPCR experiments were performed in triplicate, and error bars indicate standard deviations. Significant differences were determined using a two-tailed Student's *t*-test (**; P<0.01; ***: P<0.001). Comparisons of the Mx_WT strain with the *furA* mutant against the Sm_WT grown under the same conditions are shown at the top (green lines for *M. xanthus* and pink for *S. meliloti*); comparisons of the same strain in the same interaction grown with and without iron are shown at the bottom.
