# Supplementary material for: Siderophores and competition for iron govern myxobacterial predation dynamics
Source: ISME J. 2024 May 2;18(1):wrae077. doi: 10.1093/ismejo/wrae077 (PMC11388931; doi:10.1093/ismejo/wrae077)
Supplement: supplementary_material_wrae077 [file supplementary_material_wrae077.zip › Figure S7.pdf]

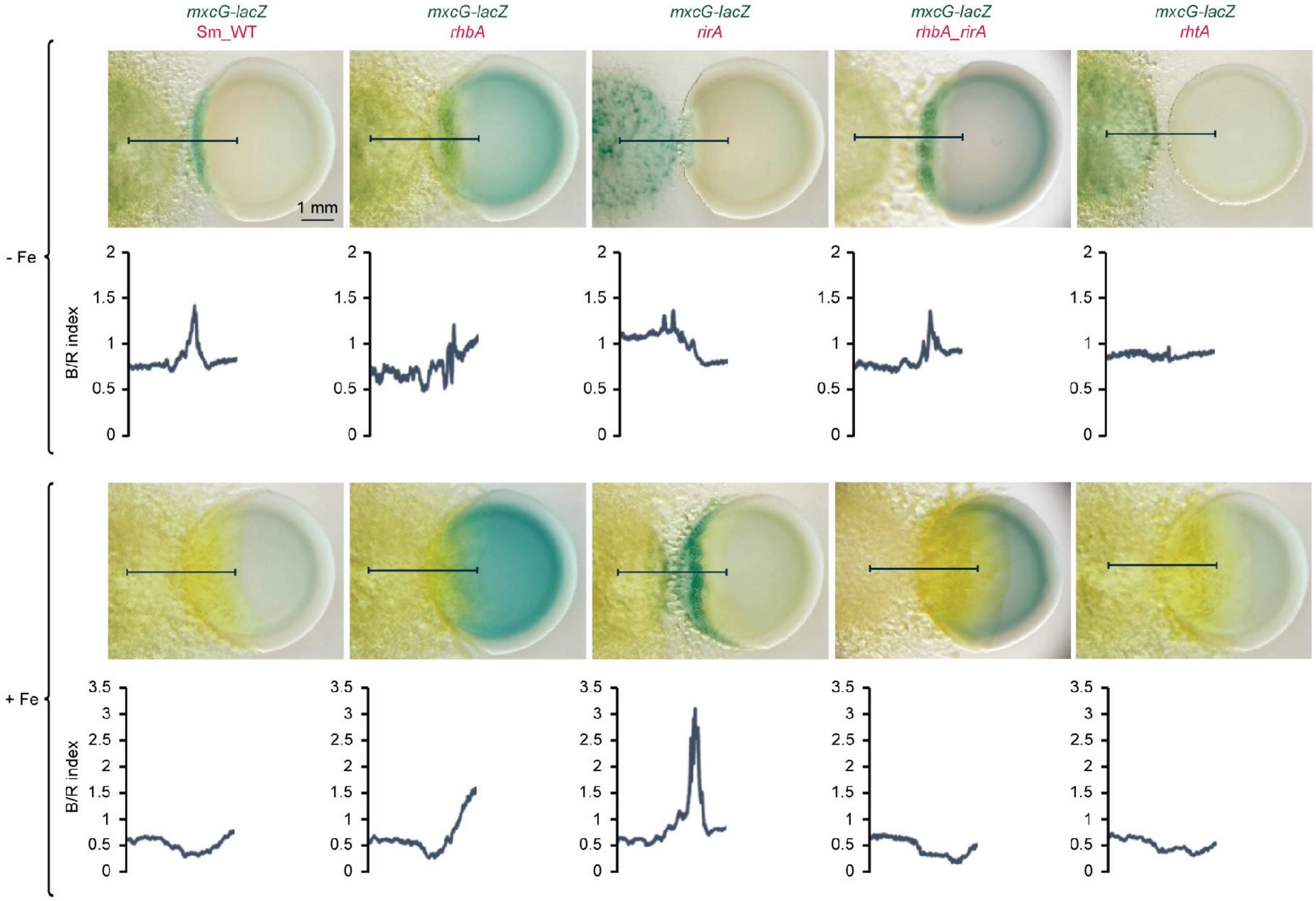

Figure S7. Expression of genes involved in myxochelin biosynthesis when the predator was confronted with the Sm_WT strain and *rhbA, rirA*, *rhbA_rirA*, and *rhtA* mutants. An *M. xanthus* strain harboring a fusion between the gene *mxcG* and *lacZ* (*mxcG-lacZ*) was assayed against the strains of *S. meliloti* mentioned above. Cells were incubated in CTT medium with or without iron supplementation and X-gal to monitor β-galactosidase activity. Pictures were taken at 72 h under a dissecting microscope with illumination from the top. Colors were scanned along the lines drawn in the pictures. To eliminate background from white light, the values shown are the ratio between blue and red (B/R index). Please note that the rhizobial *rhbA* mutant was constructed using Tn*5lac* (Table S1) and produces an intense blue color in media containing X-gal.
