# Supplementary material for: Siderophores and competition for iron govern myxobacterial predation dynamics
Source: ISME J. 2024 May 2;18(1):wrae077. doi: 10.1093/ismejo/wrae077 (PMC11388931; doi:10.1093/ismejo/wrae077)
Supplement: supplementary_material_wrae077 [file supplementary_material_wrae077.zip › Table S1.docx]

**Table S1.** Bacterial strains and plasmids used in this study.

| **Strain or plasmid** | **Relevant characteristics^a^** | **Reference or source** |
| --- | --- | --- |
| **Strains** |  |  |
| ***M. xanthus*** |  |  |
| Mx_WT (DK1622) | Wild type; Gal^r^; Km^s^ | Kaiser, 1979 |
| *mxcG* | DK1622; Δ*mxcG*; Gal^r^; Km^s^ | This study |
| *furA* | DK1622; Δ*furA*; Gal^r^; Km^s^ | This study |
| *mxcG_furA* | DK1622; Δ*mxcG* Δ*furA*; Gal^r^; Km^s^ | This study |
| *mxcG-lacZ* | DK1622; *mxcG*-*lacZ*; Km^r^ | This study |
| *furA*_*mxcG-lacZ* | DK1622 Δ*furA*; *mxcG*-*lacZ*; Km^r^ | This study |
|  |  |  |
| ***S. meliloti*** |  |  |
| Sm_WT (Rm1021) | SU47 *expR102*::IS*Rm*2011-1; Sm^r^ | Meade and Signer, 1977 |
| *rhbA* (1021rhbA) | Rm1021 *rhbA*::Tn*5lac*; Sm^r^; Nm^r^ | Nogales *et al.*, 2012 |
| 2011rhtA1 | Rm2011 *rhtA*::Tn*5*; Sm^r^ Rif^r^; Nm^r^ | Lynch *et al.*, 2001 |
| *rhtA* | Rm1021 *rhtA*::Tn*5*; Sm^r^; Nm^r^ | This study |
| *rirA* | Rm1021 Δ*rirA*; Sm^r^ | This study |
| *rhbA_rirA* | Rm1021 Δ*rirA rhbA*::Tn*5lac*; Sm^r^; Nm^r^ | This study |
| *rhb-lacZ* | Rm1021 harboring the *rhb-lacZ* fusion of plasmid pMPrhBIO; Sm^r^; Tc^r^ | This study |
| *rirA_rhb-lacZ* | Rm1021 Δ*rirA*; harboring the *rhb-lacZ* fusion of plasmid pMPrhBIO; Sm^r^; Tc^r^ | This study |
| *rhtA_rhb-lacZ* | Rm1021 *rhtA* harboring the *rhb-lacZ* fusion of plasmid pMPrhBIO; Sm^r^; Nm^r^; Tc^r^ | This study |
|  |  |  |
| ***E. coli*** |  |  |
| TOP10 | F^-^*mcrA* Δ(*mrr*-*hsdRMS*-*mcr*BC) φ80*lacZ*ΔM15 Δ*lacX*74 *recA1* *ara*D139 Δ(*ara*-*leu*)7697 *galU* *galK* *rpsL* (Str^r^) *endA1 nupG* | Invitrogen |
| DH5α | *supE44*, ∆*lacU169*, Φ80, *lacZ*∆M1, *recA1*, *endA1*, *gyrA96*, *thi1*, *relA1*, *5hsdR171* | Bethesda Research Lab^®^ |
| S17-1 | *thi*, *pro*, *recA*, *hsdR*, *hsdM*, Rp4Tc::Mu, Km::Tn*7*; Tp^r^; Sm^r^; Spec^r^ | Simon *et al.*, 1983 |
|  |  |  |
| **Plasmids** |  |  |
| pBJ113 | Cloning vector; *galK*; Km^r^ | Julien *et al*., 2000 |
| pKY481 | Cloning vector; *lacZ*; Km^r^ | Cho and Zusman, 1999 |
| pFJCMΔmxcG | Δ*mxcG*; Km^r^ | This study |
| pFJCMΔfurA | Δ*furA*; Km^r^ | This study |
| pFJCMmxcGlacZ | *mxcG*-*lacZ*; Km^r^ | This study |
| pK18mobsacB | Suicide plasmid; Km^r^ | Schäfer *et al.*, 1994 |
| pK18-ΔrirA | pK18mobsacB carrying the deleted version of the *rirA* locus; Km^r^ | This study |
| pMP220 | IncP transcriptional fusion vector; Tc^r^ | Spaink *et al.*, 1987 |
| pMPrhBIO | pMP220 derivative carrying a transcriptional fusion of the promoter for the rhizobactin 1021 biosynthesis operon to *lacZ* (*rhb-lacZ*); Tc^r^ | This study |

^a^ Sm^r^, Nm^r^, Rif^r^, Tp^r^, Spec^r^, Km^r^, Tc^r^, and Gal^r^, and: streptomycin, neomycin, rifampin, trimethoprim, spectinomycin, kanamycin, tetracycline, and galactose resistance, respectively; Km^s^: kanamycin sensitive.

**REFERENCES**

Cho, K., and Zusman, D.R. (1999). AsgD, a new two-component regulator required for A-signalling and nutrient sensing during early development of *Myxococcus xanthus*. *Mol. Microbiol*, 34:268-281.

Julien, B., Kaiser, A.D., and Garza, A. (2000). Spatial control of cell differentiation in *Myxococcus xanthus*. *Proc. Natl. Acad. Sci. U.S.A.*, 97:9098-9103.

Kaiser, D. (1979). Social gliding is correlated with the presence of pili in *Myxococcus xanthus*. *Proc. Natl. Acad. Sci. U.S.A.*, 76:5952-5956.

Lynch, D., O'Brien, J., Welch, T., Clarke, P., Cuiv, P.O., Crosa, J.H., and O'Connell, M. (2001). Genetic organization of the region encoding regulation, biosynthesis, and transport of rhizobactin 1021, a siderophore produced by *Sinorhizobium meliloti*. *J. Bacteriol.*, 183:2576-2585.

Meade, S.M., and Signer, E.R. (1977). Genetic mapping of *Rhizobium meliloti*. *Proc. Natl. Acad. Sci. U.S.A.*, 74:2076-2078.

Nogales, J., Bernabéu-Roda, L., Cuéllar, V., and Soto, M.J. (2012). ExpR is not required for swarming but promotes sliding in *Sinorhizobium meliloti*. *J. Bacteriol.*, 194:2027-2035.

Schäfer, A., Tauch, A., Jager, W., Kalinowski, J., Thierbach, G., and Pühler, A. (1994). Small mobilizable multi-purpose cloning vectors derived from the *Escherichia coli* plasmids pK18 and pK19: selection of defined deletions in the chromosome of *Corynebacterium glutamicum*. *Gene*, 145:69-73.

Simon, R., Priefer, U., and Pühler, A. (1983). A broad host range mobilization system for *in vivo* genetic-engineering: transposon mutagenesis in Gram-negative bacteria. *Nat. Biotechnol.*, 1:784-791.

Spaink, H.P., Okker, R.J., Wijffelman, C.A., Pees, E., and Lugtenberg, B.J. (1987). Promoters in the nodulation region of the *Rhizobium leguminosarum* Sym plasmid pRL1JI. *Plant Mol. Biol.*, 9:27-39.
