# Supplementary material for: Siderophores and competition for iron govern myxobacterial predation dynamics
Source: ISME J. 2024 May 2;18(1):wrae077. doi: 10.1093/ismejo/wrae077 (PMC11388931; doi:10.1093/ismejo/wrae077)
Supplement: supplementary_material_wrae077 [file supplementary_material_wrae077.zip › Table S2.docx]

**Table S2.** Primers and probes used in this study.

| **Primer** | **Sequence (5´ to 3´)^a^** | **Used for** |
| --- | --- | --- |
| MXAN3643lacBamHI | CGTGGATCCATGCGTCAGGGGCGCGAGGT | Amplification of the upstream region of *mxcG* to obtain the strain *mxcG*-*lacZ* |
| MXAN3643lacKpnI | GTGGGTACCTTCACGCAGGGCCAGTCGCC |  |
|  |  |  |
| MXAN3643HindIII | CTCAAGCTTACGCAGGGCCAGTCGCCCGT | Amplification of the upstream region of *mxcG* to obtain the *mxcG* mutant |
| MXAN3643BamHI | CCGGGATCCCATGCGTCAGGGGCGCGAGG |  |
|  |  |  |
| MXAN3643BamHI2 | GCAGGATCCTGACGAACCCCACCTGGACG | Amplification of the downstream region of *mxcG* to obtain the *mxcG* mutant |
| MXAN3643EcoRI | GAGGAATTCGGTGCGCTCGCCAATCCACA |  |
|  |  |  |
| MXAN3702HindIII | CGAAAGCTTCCTTGACGGTGGCCTCCAGG | Amplification of the upstream region of *furA* to obtain the *furA* mutant |
| MXAN3702BamHI1 | GGTGGATCCCATGCCTCGGGCGCACTTGG |  |
|  |  |  |
| MXAN3702BamHI2 | TCGGGATCCTGATCTCCATGTCCCGCGTG | Amplification of the downstream region of *furA* to obtain the *furA* mutant |
| MXAN3702EcoRI | CGCGAATTCATCCTTGCGCAGTGCGGCAA |  |
|  |  |  |
| gapAF | CAAGGAAGACCTCGAGATTG | ddPCR quantification of *M. xanthus*. Coordinates of the *gapA* gene are 3287094..3288104, representing a position of 71.9% of half chromosome |
| gapAR | GAGTCGTACTTGAACAGGTG |  |
|  |  |  |
| gapAI | [6FAM]TCGACAAGCCCGCGGCGCTG[BHQ1] | Probe for ddPCR quantification of *M. xanthus* |
|  |  |  |
| rplMF | CGTCATCGTCATCAATGCAG | ddPCR quantification of *S. meliloti.* Coordinates of the *rplM* gene are 1350749..1351213, representing a position of 73.9% of half chromosome |
| rplMR | CCAGTAGTACTTCTTGTCGG |  |
|  |  |  |
| rplMI | [HEX]AAGGCCGTCCTCACCGGCAAGAAGT[BHQ1] | Probe for ddPCR quantification of *S. meliloti* |
|  |  |  |
| SmRirA1 | TTAAGCTTggaacagaaccttgatcg | *rirA* deletion |
| SmRirA2 | GAGGAAGTTGATCTGGGTTTGCTTCGTCAG |  |
| SmRirA3 | CTGACGAAGCAAACCCAGATCAACTTCCTC |  |
| SmRirA4 | AATCTAGAGATCTTGACCGTCATGTC |  |
|  |  |  |
| RhbF | TTTGAATTCATCATGATCTTCGACGCTGC | Amplification of the *rhtXrhbABCDEF* promoter region |
| RhbR | TTTCTGCAGAGCATCGAAAACTGCCACTG |  |

^a^ Restriction sites used for cloning are underlined and fluorescent dyes of the probes are enclosed in square brackets.
